# Prioritizing isolation precautions: a patient-centered approach to infection prevention and control

**DOI:** 10.1017/ash.2025.173

**Published:** 2025-06-03

**Authors:** Emine Alp Meşe, Elena Carrara, Ermira Tartari, Nico T. Mutters, Constantinos Tsioutis, Gabriel Birgand, Evelina Tacconelli

**Affiliations:** 1 ESCMID European Committee on Infection Prevention and Control (EUCIC), Basel, Switzerland; 2 Department of Infectious Diseases and Clinical Microbiology, Faculty of Medicine, Ankara Yıldırım Beyazıt University, Ankara, Türkiye; 3 Division of Infectious Diseases, Department of Diagnostic and Public Health, University of Verona, Verona, Italy; 4 Faculty of Health Sciences, Mater Dei Hospital, Msida, Malta; 5 Institute for Hygiene and Public Health, University Hospital Bonn, Bonn, Germany; 6 School of Medicine, European University Cyprus, Nicosia, Cyprus; 7 Regional Center for Infection Prevention and Control, Region of Pays de la Loire, Nantes University Hospital, Nantes, France; 8 National Institute for Health Research Health Protection Research Unit in Healthcare Associated Infections and Antimicrobial Resistance, Imperial College London, London, UK; 9 Cibles et médicaments des infections et de l’immunité, IICiMed, Nantes Université, Nantes, France; 10 Infectious Diseases Section, Department of Diagnostics and Public Health, University of Verona, Verona, Italy

## Abstract

Healthcare-associated infections (HAIs) and multidrug-resistant (MDR) pathogens present significant challenges to global health, exacerbated by emerging threats such as SARS-CoV-2 and the growing immunocompromised population. While isolation precautions are critical for infection prevention and control (IPC), their indiscriminate application can strain resources and impact patient well-being. This review proposes a patient-centered framework for optimizing isolation strategies by integrating pathogen-related factors, individual patient risks, and healthcare facility resources to optimize isolation precautions. By incorporating targeted risk assessments, advanced analytics (e.g., omics and machine learning), and infection preventionist leadership, this approach aligns isolation measures with clinical and operational realities. It aims to enhance IPC efficacy while balancing patient needs and resource efficiency. We highlight strategies to ensure isolation precautions remain evidence-based, adaptable, and sustainable within healthcare settings. A patient-focused approach to isolation improves both infection prevention and overall quality of patient care.

## Introduction

Healthcare-associated infections (HAIs) pose a major challenge to healthcare systems globally, increasing morbidity, mortality, and economic burden.^1,2^ Isolation precautions play a critical role in reducing transmission; however, their indiscriminate application can strain resources, compromise patient experience, and lead to unintended consequences such as increased workload for healthcare workers and psychological distress for patients.^3–6^

A key limitation of current infection prevention and control (IPC) strategies is the lack of personalized risk stratification for patient isolation. Traditional protocols adopt a one-size-fits-all approach, which may not always align with the specific risks posed by different pathogens, patient conditions, or healthcare facility constraints.^7^ At the same time, emerging multidrug-resistant (MDR) bacteria, novel viral pathogens, and the growing immunocompromised population necessitate more efficient and targeted isolation strategies.^4^

This review aims to develop a patient-centered approach to prioritizing isolation precautions, ensuring that interventions are both effective in infection control and minimally disruptive to patient care. We propose an integrated framework that considers:

1. Patient risk factors – underlying health conditions, omics-based susceptibility, and colonization risk.
2. Pathogen-related factors – transmission potential, virulence, and environmental persistence.
3. Healthcare facility constraints – availability of isolation rooms, workload pressures, and adherence challenges.

By incorporating advanced analytics (e.g., machine learning, omics-based risk assessments) and infection prevention leadership, this model seeks to optimize the use of isolation resources while maintaining high-quality patient care.

This paper first examines the challenges in implementing isolation precautions, followed by a discussion of key patient, pathogen, and facility-related factors influencing prioritization. By integrating a patient-centered approach into IPC strategies, we aim to provide a more adaptive, evidence-based, and resource-efficient model for prioritizing isolation precautions in modern healthcare settings. This approach aims to enhance decision-making in isolation practices, supported by emerging technologies such as AI-driven risk stratification and omics-based infection risk profiling.

### Importance of isolation precautions in IPC

In healthcare facilities, MDR bacteria can be transmitted through various routes, including direct contact with contaminated surfaces, exposure to contaminated medical equipment, and person-to-person transmission among patients and healthcare workers.^8^ Isolation of patients who are colonized or infected with MDR pathogens is the mainstay of IPC precautions.^3^

On the other hand, the world has witnessed a concerning rise in the incidence of emerging and re-emerging pathogens, including the notable example of SARS-CoV-2, the virus responsible for the COVID-19 pandemic in recent years. Isolation precautions are critical for managing emerging and re-emerging pathogens, which pose significant threats to global health. These pathogens, often characterized by their ability to spread rapidly and cause severe illness, require stringent isolation measures to prevent transmission within healthcare settings and the community.^4^

At the same time, limited knowledge of transmission routes for emerging pathogens during the early stages in hospital settings, combined with insufficient personal protective equipment (PPE), greatly increases the risk of infection among healthcare workers (HCWs). During the initial phase of the COVID-19 pandemic, a study conducted in Sweden (March–May 2020) found that 33% of HCWs in an inpatient ward acquired COVID-19. Among them, 96% had direct patient contact, and 78% were linked to exposure from infectious colleagues, while community and household transmission were less frequent.^5^

The significance of isolation precautions cannot be overstated as they play a critical role for mitigating the transmission of infectious agents and safeguarding patients, healthcare workers, and visitors.

### Challenges with one-size-fits-all isolation strategies

Despite the critical need for isolation rooms in healthcare settings, the rising prevalence of MDR pathogens in both hospitals and communities, along with emerging and re-emerging pathogens and a growing number of immunocompromised patients, poses significant challenges for healthcare facilities. The demand for isolation rooms often exceeds the supply, particularly in developing and Eastern European countries, where limited staffing and inadequate availability of isolation rooms leads to low adherence to isolation precautions.^9–11^ The ECDC Surveillance Report for 2022–2023 highlighted the differences in hospital infrastructure across European acute care hospitals. The median proportion of single-bed rooms was below 5% in Greece, Hungary, Romania, Kosovo, Montenegro, and Serbia, and exceeded 50% in France and Sweden. Meanwhile, the median number of airborne infection isolation rooms was 16.0 per 1,000 hospital beds, ranging from fewer than one isolation room per 1,000 beds in Hungary, Montenegro, and Serbia to 30 or more per 1,000 beds in Finland, Italy, and Sweden.^12^

Conversely, isolation rooms may negatively affect patient care. Patients may experience loneliness, anxiety, depression and reduced interactions with the staff and family. Healthcare workers face an increased workload and risk of burnout due to additional precautions. Limited access to family members can cause emotional distress, and strict protocols may lead to errors or cross-contamination.^13–15^

In this context, there is a global need to adopt a personalized approach for prioritizing patient admission to isolation rooms, considering clinical, epidemiological, and institutional factors. Effective prioritization of isolation precautions is essential to enhance their efficacy while minimizing the negative impacts of IPC measures, such as costs, increased workload, and psychological effects.

### Factors influencing the prioritization of isolation precautions

Patient-centered infection control offers a transformative approach to infection prevention by emphasizing tailored strategies that account for individual patient characteristics, emerging microbial threats and healthcare facility-specific factors. This approach moves beyond traditional one-size-fits-all protocols, aiming for more rationalized use of facilities and opportunities and better patient outcomes.^7^ This is further supported by the fact that specific drivers can influence human-to-human transmission.^16^

Savoldi *et al*.^13^ outlined the core principles of personalized IPC, which include patient risk assessment (colonization, underlying diseases, and omics profile), pathogen-related factors (molecular resistance mechanisms, plasmids, and virulence factors), and facility characteristics (epidemiology, infrastructure, healthcare workload and surveillance). The decision to prioritize patients for isolation requires careful consideration of multiple factors, and infection preventionists play a critical leadership role in evaluating these factors to make informed and effective isolation decisions.

## A framework for patient-centered isolation prioritization

### Patient risk factors

#### Omics-based susceptibility analysis

Prioritization of patients for placement in isolation rooms can be significantly enhanced through patient risk assessment and integration of omics technologies that provide a deeper understanding of individual susceptibility and transmission risks. Advancements in omics technologies have revolutionized the field of medical research, offering profound insights into the complex interactions between hosts and pathogens. In the study of colonization and infection, patient omics, including genomics, transcriptomics, proteomics, metabolomics, and microbiomics, can offer a detailed view of biological processes. These insights pave the way for personalized medicine approaches that can improve prevention, diagnosis, and treatment for infectious diseases.^17^ Developments in these technologies have transformed personalized IPC by allowing the identification of host genetic factors that affect susceptibility to infection and the risk of transmission. By customizing isolation measures according to individual omics profiles, isolation strategies can be more precise and effective. Omics data allow for the identification of host genetic factors that influence susceptibility to infections and individual responses to treatments. This facilitates personalized medicine approaches, where interventions can be tailored based on a person’s genetic predispositions and microbial profiles.^18,19^ However, there is a gap in the literature regarding association between patient omics and pathogen acquisition, and further studies are needed to address this issue.

#### Underlying health conditions and colonization risk

Factors such as increased colonization sites, advanced age, morbidities, previous antibacterial therapy, cancer chemotherapy, previous gastrointestinal surgery, prior healthcare exposure including in long-term facilities and previous detection of MDR pathogens further increase the risk of colonization.^20^ A hospital-based case-control study identified liver cirrhosis, previous MDR-GNB carriage, digestive surgery, and length of hospital stay within the previous year as independent risk factors for MDR-GNB colonization. However, only liver cirrhosis had a strong association with MDR colonization.^21^

Hu *et al*.^22^ demonstrated that continuous carriage extends the shedding of carbapenem-resistant *Klebsiella pneumoniae* (CRKP) into the environment, leading to persistent contamination. Additionally, a prolonged intensive care unit (ICU) stay (>6 weeks) increases interactions with other patients and HCWs, thereby expanding contamination and facilitating the spread of bacterial strains. These index patients can act as “super-contaminators,” driving extensive and persistent environmental contamination and amplifying the spread of their CRKP clones, suggesting that isolating “super-contaminators” should be a priority. In a study by Lerner *et al*.,^23^ 18% of carriers accounted for 80% of environmental contamination, with individuals having high rectal concentrations of KPC-producing CRE being significantly more likely to act as super-spreaders compared to non-super-spreaders. Additionally, the absence of fecal incontinence was the only variable significantly associated with being a non-spreader.

In terms of patient-related factors, isolation should be prioritized for individuals identified as super-contaminators, particularly those with symptomatic infections and multiple sites of colonization. High-risk groups include immunocompromised patients, those with elevated bacterial or viral loads, individuals with diarrhea, and patients undergoing extensive medical interventions or invasive procedures such as catheterization, mechanical ventilation, or surgery.^24,25^

### Pathogen-related factors

#### Transmissibility and mode of transmission

Pathogen transmissibility is a key determinant in selecting appropriate isolation precautions. Highly infectious pathogens require stringent measures in healthcare settings due to their potential for rapid spread, from one host to another, and large-scale outbreaks, often leading to severe disease. Pathogens that travel through air often exhibit higher transmission rates than those requiring direct contact. Additionally, pathogens with prolonged environmental survival pose an increased risk of transmission.^26^ Transmission rates, commonly expressed as the basic reproduction number (R), are influenced by factors such as modes of transmission, environmental stability, population density, infection control measures, and immunization coverage.^27^ High vaccination coverage contributes to herd immunity, limiting the spread of highly contagious diseases. In contrast, emerging pathogens lacking herd immunity, such as SARS-CoV2, require stringent isolation precautions and prioritization.^28,29^ Additionally, the rapid horizontal transfer of AMR genes via plasmids and transposons among enteric bacteria facilitates the persistence and dissemination of AMR.^30^ Key drivers of transmission include specific resistance genes (e.g., ESBL, carbapenemase genes), high-risk clones such as *K. pneumoniae* ST147 and ST307, and genomic factors that enhance pathogen virulence and transmissibility.^31^

The transmission rates of MDR pathogens can differ, and different mechanisms may drive patient-to-patient transmission chains. Studies have shown that ESBL-producing *K. pneumoniae* is an ESBL-producing *Escherichia coli*. In relation to bacterial factors, studies have demonstrated that environmental contamination occurs more frequently around patients colonized with *Klebsiella* spp. than those colonized with *E. coli*. These studies indicate that *Klebsiella* spp. can persist in the environment for extended periods because of their biofilm-forming ability. This environmental contamination facilitates the intra-hospital spread of *Klebsiella* spp., explaining their higher rates of cross-transmission and increased potential for outbreaks.^32–34^ Person-to-person transmission of certain ESBL-producing *E. coli* is not a frequent occurrence in most hospital environments.^35–38^ In a prospective multicenter study that included molecular genetic analyses assessing the impact of single-room contact precautions (SCP) on the hospital acquisition and transmission of *E. coli* resistant to third-generation cephalosporins (3GCR), with screening for intestinal colonization with 3GCR-*E. coli* using deep rectal swabs or stool samples within 72 hours of admission, weekly, and within 72 hours of discharge and bloodstream infection, no advantage was found for SCP compared to no contact precautions in preventing 3GCR-*E. coli* hospital acquisition and patient-to-patient transmission in a high-risk setting of hematological and oncological patients.^39^ To this end, previous studies have reported that controlling ESBL-*Enterobacterales* entails far more difficulties compared to strategies targeted towards MRSA, owed to their different microbiological characteristics and epidemiology that facilitate environmental persistence and transmission.^40^

In cases of isolation room shortages, de-escalation of isolation precautions can be guided by pathogen transmissibility. Chang *et al*.^41^ modified isolation protocols for patients colonized or infected with vancomycin-resistant *Enterococci* (VRE) following an observed increase in the incidence of HAIs caused by MDR, including carbapenem-resistant Enterobacterales (CRE). During the study period, VRE-positive patients were not isolated or cohorted, while hand hygiene, contact precautions (e.g., gloves and gowns), and environmental disinfection protocols remained unchanged. The study found no significant change in VRE bacteremia incidence with the relaxation of isolation measures, provided that other IPC measures were consistently implemented. Although some studies have reported an association between discontinuation of contact precautions and active screening programs for VRE and increased rates of VRE bacteremia,^42^ recent data suggest that contact precautions can be safely discontinued without a rise in MRSA or VRE infections. A survey by the Society for Healthcare Epidemiology of America Research Network (SRN), a consortium of acute care facilities, showed that while only 7% of SRN hospitals had discontinued using routine contact precautions in 2015, this figure increased to 35% by 2021. More than 90% of facilities that discontinued contact precautions cited scientific evidence as the primary rationale for their decisions.^43^ The decision to discontinue contact precautions should consider pathogen-related factors (e.g., surveillance samples, duration of colonization), patient-specific characteristics (such as symptoms, antibiotic use, length of hospital and intensive care unit stays), and institutional risk levels. Infection prevention and control leadership must regularly review and update policies, especially when there are changes in the epidemiology of concerning pathogens, such as during outbreaks or hyperendemic situations.^44,45^ Further research is still needed to confirm the safety of discontinuing contact precautions, explore alternative strategies for their implementation, and address potential patient harms in settings where they remain in use.

#### Environmental persistence and outbreak potential

Pathogens that can survive in environmental conditions for long periods of time pose a significant outbreak risk because they can persist on surfaces and equipment and facilitate transmission in healthcare environments.

*Acinetobacter* spp. is a common cause of HAIs with increasing frequency of MDR in recent years. Numerous outbreaks have been reported because of high environmental contamination rates and the ability for prolonged survival at these sites.^46,47^ *Acinetobacter* spp. are known to persist on environmental surfaces for extended periods lasting several weeks.^48^ Additionally, studies have demonstrated the airborne dispersal of MDR *A. baumannii*.^49^ In a study by Wong *et al*.,^50^ they prioritized COVID-19 cases for isolation rooms during the pandemic and used open cubicles for patients with MDR *A. baumannii.* They implemented contact precautions, active screening of all hospitalized patients, cohort nursing, and environmental disinfection for new cases of MDR *A. baumannii* infections. Environmental and air samples were collected for MDR *A. baumannii* culture, and nosocomial transmission of *A. baumannii* was described. Their findings concluded that MDR *A. baumannii* could be dispersed through the air, as it was detected in air samples and contaminated hard-to-reach surfaces. They strongly recommended single-room isolation or the addition of a portable high-efficiency particulate air (HEPA) filter to conventionally designed open cubicles, particularly when the cubicles are fully occupied by patients with MDR *A. baumannii*. Moreover, they suggested that hospital renovation and redevelopment programs should include the installation of an air ventilation system with self-contained air inflow and exhaust within each cubicle, preferably with the door closed. Other studies have also shown that MRSA and carbapenemase-producing *Enterobacterales* can be spread through the air, highlighting the necessity for targeted measures to reduce the risk of airborne transmission in healthcare facilities.^51^

*Clostridioides difficile* and n*orovirus* are other common pathogens that can cause nosocomial outbreaks owing to environmental contamination. Owing to their ability to survive for prolonged periods on surfaces, environmental contamination plays an important role in their transmission in healthcare institutions and can thus be considered a priority.^48^ Although contact precautions are generally recommended to continue for the duration of illness in *C. difficile* infections and for at least 48 hours after symptom resolution or to manage institutional outbreaks in norovirus, a personalized approach may be appropriate for extending these precautions. Such an approach should consider patients with ongoing risk factors for colonization and/or contamination, such as advanced age, antibiotic use, incontinence, immunosuppression.^52,53^

Several factors influence the survival of bacteria in the environment. Although the type of bacterium and its inoculum size are key factors for survival, the material of the surface also plays a significant role in determining how long bacteria can persist. Neely^54^ conducted a study on seven common gram-negative nosocomial bacteria and tested their survival on seven different materials. The findings revealed that the bacteria tended to survive longer on synthetic surfaces than on cotton, and even longer on plastic surfaces than on fabrics. Additionally, bacterial viability varied depending on the microorganism, with *Pseudomonas* surviving for a shorter duration than *Enterobacterales* and *Acinetobacter spp.* However, another experimental study that quantitatively investigated the impact of environmental conditions on the survival of 60 healthcare-associated MDR bacteria, including MRSA, VRE, *K. pneumoniae*, and *A. baumannii*, reported controversial results. Factors such as textile type, presence of nutrients, temperature, and humidity were examined in this study. The researchers found that Gram-positive MRSA and VRE had significantly higher survival rates on polyester compared to cotton, while Gram-negative MDR *Acinetobacter* spp showed significantly lower survival on polyester than on cotton. Additionally, MDR *K.pneumoniae* and MRSA survival rates increased significantly in nutrient broth compared to the control conditions, whereas the presence of nutrients did not significantly affect the of MDR *Acinetobacter* spp and VRE.^55^ Understanding how different environmental conditions affect the survival of Gram-positive and Gram-negative bacteria is valuable for isolation measures and identifying outbreak sources.

Therefore, isolation precautions for multidrug-resistant (MDR) pathogens should be determined according to patient-specific factors, the types of procedures (i.e., small particle-forming procedures) performed, and the specific characteristics of the healthcare facility.

### Healthcare facility constraints

#### Hospital capacity and infrastructure

Facility characteristics are key determinants of the effectiveness of isolation measures. These include infrastructure, design, equipment, staffing levels, surveillance/screening strategies, and the local epidemiology of pathogens. Transmission rates of identical bacteria can vary depending on staff workload, facility design and infrastructure, screening strategies, antimicrobial stewardship, and patient population. Architectural features such as the separation of clean and contaminated areas, strategic patient flow, proper ventilation system in wards and isolation rooms with negative pressure ventilation are critical to prevent cross-contamination and controlling infectious agents, thus improving overall IPC outcomes.^56^

## Risk-based stratification & implementation strategies

### Risk categories for isolation precautions

**Figure 1.**
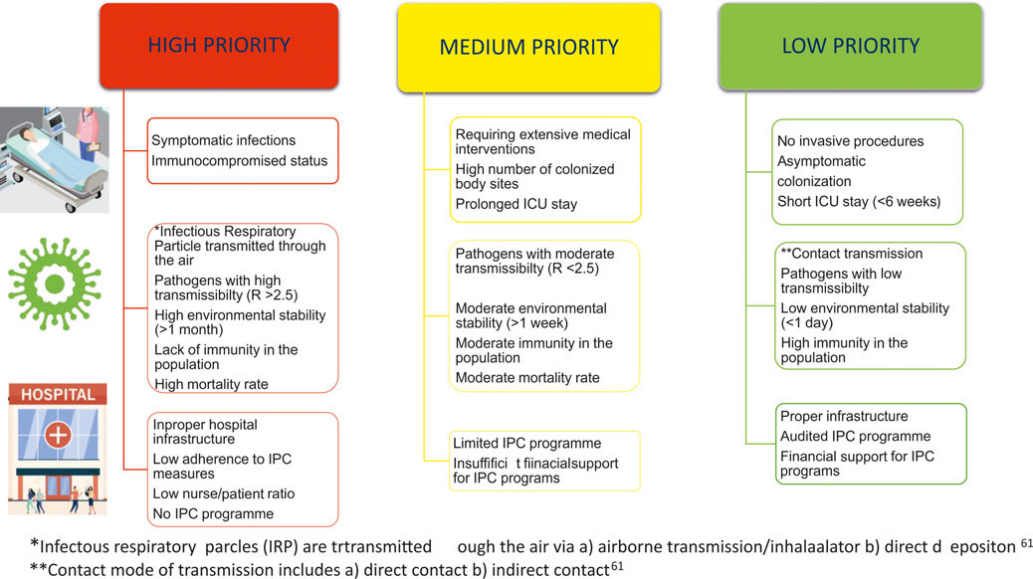
*Infectous respiratory parcles (IRP) are trtransmitted ough the air via a) airborne transmission/inhalaalator b) direct depositon.^61^ **Contact mode of transmission includes a) direct contact b) indirect contact.^61^

Isolation prioritization is a key strategy in IPC, focusing on categorizing patients into high-risk, medium-risk, and low-risk groups based on patient factors, pathogen characteristics, and healthcare facility constraints (Figure 1). High-risk patients include those with symptomatic infections, immunocompromised status, and exposure to highly transmissible pathogens (*R* > 2.5) with high environmental stability and mortality rates. These patients require immediate isolation with strict precautions, especially in facilities with poor infrastructure, low IPC adherence, or insufficient staffing. Medium-risk patients, such as those requiring extensive medical interventions, prolonged ICU stays, or colonized at multiple sites, are exposed to moderately transmissible pathogens (*R* < 2.5) with moderate environmental stability and mortality rates. They need isolation with standard precautions and regular monitoring, particularly in facilities with limited IPC programs or funding. Low-risk patients, including asymptomatic individuals, those without invasive procedures, or with short ICU stays, are exposed to low-transmissibility pathogens that have low environmental stability and high population immunity. These patients require minimal isolation measures, especially in facilities with proper infrastructure, audited IPC programs, and adequate funding. By prioritizing isolation based on these risk categories, healthcare facilities can optimize resource allocation, implement targeted IPC measures, and effectively reduce the risk of healthcare-associated infections.

### Role of AI & machine learning in IPC

Machine learning, a branch of artificial intelligence (AI), focuses on developing algorithms and models that enable computers to learn from data and make predictions, analyze a variety of variables in electronic health records to go beyond traditional HAI risk stratification, and create custom models for specific facilities or populations. It adapts to dynamic healthcare changes and alerts IPC teams to evolving patient risks.^57,58^ Although further research is needed, this approach has the potential to revolutionize HAI surveillance and IPC by identifying high-risk patients and optimizing treatment. In addition, AI improves IPC processes by improving hand hygiene compliance and implementation. Integrating machine learning into IPC strategies enables the rapid identification of high-risk patients. Predictive models can process complex, patient-centralized data to predict which individuals require immediate isolation, optimize resource allocation, and improve patient outcomes. By leveraging these models, healthcare facilities can more effectively identify potential “super-contaminators” early on, allowing for more targeted IPC measures. However, most studies evaluating AI in IPC are retrospective, highlighting the need for prospective evaluations in real-world, often unpredictable clinical settings. The success of AI relies on high-quality, comprehensive data, well-defined reference standards (often lacking in IPC), and strong collaboration with IPC experts to interpret findings and ensure clinical relevance. Without these elements, machine-learning models can introduce errors and lead to false negatives, misclassifications, or limited applicability. Additionally, IPC practitioners must be aware of the limitations of AI, including underfitting (poor classification of new data), overfitting (difficulty recognizing similar patterns in new data), and potential biases in the training data.^59^

### Environmental controls and disinfection strategies

Environmental contamination plays a crucial role in the transmission of infections within healthcare settings, as patient surroundings often serve as reservoirs for cross-contamination, leading to colonization and subsequent infection.^15^ The recent Global Technical Consultation Report by the World Health Organization (WHO) defines “infectious respiratory particles (IRPs)” as airborne particles that contain pathogens. The report emphasizes that several environmental factors—including temperature, humidity, air velocity, ultraviolet radiation, and airflow distribution within enclosed spaces—affect the dispersion and transmission of IRPs, as well as their viability and infectivity upon reaching other individuals.^60^ Consequently, these findings have direct implications for the design and implementation of isolation precautions. Therefore, maintaining optimal temperature and humidity within the hospital environment is critical for minimizing microbial growth and transmission. Advanced ventilation systems, including proper air filtration, regular air exchanges, and humidity control, can significantly reduce the spread of infections, such as tuberculosis and COVID-19. Technologies such as HEPA filters and ultraviolet germicidal irradiation (UVGI) further enhance air quality and reduce microbial loads.^61,62^ Raggi *et al*.^63^ evaluated the effectiveness of UV-C terminal disinfection in reducing HAIs caused by MDR bacteria in a community hospital setting. Using a pre-post study design, this study analyzed HAI rates, emergency room wait times, and cost savings over a 12-month period. The results demonstrated a 19.2% reduction in HAIs (from 4.87 to 3.94 per 1,000 patient days; *P* = .006), no negative impact on emergency department wait times (297.9 vs 296.2 minutes; *P* = .18), and direct cost savings of $1,219,878. Despite the need for further studies to confirm its clinical effectiveness and identify optimal implementation strategies, UV disinfection represents a promising solution for resource-constrained healthcare systems, offering a potential pathway to reduce infections and improve patient outcomes.

### Patient engagement strategies

Patient engagement plays a critical role in ensuring adherence to personalized isolation measures. Educating patients about their unique risk factors and involving them in decision-making not only improves adherence to these isolation measures but also fosters a sense of responsibility and engagement in their own care. This partnership between patients and healthcare providers is essential, particularly for those identified as high-risk or through predictive modeling, as active involvement in isolation strategies can significantly reduce environmental contamination and the spread of infections.^64^

### Policy in resource limited countries

In resource-limited countries, the main challenges in IPC arise from inadequate government regulations, a lack of support and guidance for building effective IPC infrastructure, and limited financial resources to establish strong IPC programs and supply of PPE. These constraints lead to poor adherence to isolation measures, contributing to the endemic spread of MDR pathogens in hospitals.^65^ Without effective isolation measures, MDR pathogens become endemic in these hospitals, continuing the cycle of transmission. In settings where MDR pathogens are endemic, IPC measures may differ from those applied during epidemics.^36^ In endemic settings, when isolation and cohorting are impractical due to barriers such as high colonization pressure, open-plan ICUs, insufficient bed separation, lack of isolation rooms, and staff shortage, applying universal contact precautions can effectively prevent the horizontal spread of pathogens in ICUs with severe disease conditions and high colonization pressures.^66,67^ In resource-limited settings where multidrug-resistant bacteria are endemic, each healthcare facility should determine its own IPC policies in terms of isolation priority of microorganisms (eg*. Acinetobacter* vs. ESBL-producing *E.coli*) and rationale use of PPE, alternative PPE strategies (eg.reusable gowns vs. disposable plastic) and strategically allocate resources to effectively address the challenges.

## Conclusion

The growing demand for isolation rooms in healthcare facilities, driven by the increasing prevalence of MDR bacteria, emerging pathogens, and a rising population of immunosuppressed individuals, highlights the critical need to prioritize effective isolation precautions. This prioritization must consider key factors, including effective microorganism characteristics (e.g.,transmissibility, infectivity, environmental contamination, survival on surfaces, tenacity), patient-specific aspects (e.g., susceptibility, microbial load, and omics profiles), and institutional infrastructure and resources (e.g., availability of isolation rooms, adherence to IPC practices, hand hygiene practices, staff workload, and staff levels).

Effective prioritization also relies on systematically addressing challenges to implementation within healthcare systems, including staff training, resource allocation, institutional culture, and communication processes. These factors are crucial for ensuring that isolation precautions are applied where they are most needed and can achieve the greatest impact. Although further research is required to strengthen the evidence linking these factors to optimal IPC strategies, the current challenges posed by rapidly evolving pathogens and vulnerable patient populations demand a comprehensive and well-structured approach to prioritizing isolation measures.

## Future directions

Emerging technologies such as AI, omics, and UV disinfection provide promising opportunities to advance infection prevention and control (IPC) practices and achieve better patient outcomes.

More research is needed to validate AI applications in IPC, explore omics-based risk assessments, and evaluate the long-term benefits of advanced technologies like UV disinfection, especially in resource-limited areas. Healthcare leaders play a key role in implementing these strategies, and there is a strong call for international standardization of IPC practices to ensure consistency and effectiveness worldwide.

By focusing on these areas and promoting collaboration among researchers, policymakers, and healthcare providers, we can strengthen IPC efforts, reduce healthcare-associated infections, and create safer environments for patients and healthcare workers alike.
